# Treatment of Dietary Defatted *Hermetia illucens* Larvae Meal With Different Doses of γ‐Rays: Effects on Growth, Waste Production, Antioxidant Capacity, and Gut Microbiota in *Acanthopagrus schlegelii*

**DOI:** 10.1155/anu/7228323

**Published:** 2026-01-15

**Authors:** Yubo Wu, Xiaojie Lu, Yueyuan Tan, Tinglong Yang, Jinxing Zhu, Jie Wang, Xiujuan Wang, Xing Ren

**Affiliations:** ^1^ Zhejiang Provincial Key Laboratory of Plant Evolutionary Ecology and Conservation, School of Life Sciences, Taizhou University, Taizhou, 318000, Zhejiang, China, tzc.edu.cn; ^2^ Key Laboratory of Tropical Marine Ecosystem and Bioresource, Guangxi Key Laboratory of Beibu Gulf Marine Resources, Environment and Sustainable Development, Fourth Institute of Oceanography, Ministry of Natural Resources, Beihai, 536000, Guangxi, China, mnr.gov.cn; ^3^ Linhai Fisheries Technology Extension Center, Taizhou, 318000, Zhejiang, China; ^4^ Sanmen Donghang Aquatic Seedling Technology Co., Ltd., Taizhou, 317111, Zhejiang, China

**Keywords:** *Acanthopagrus schlegelii*, antioxidant enzymes, gamma ray irradiation, *Hermetia illucens*, intestinal microbiota

## Abstract

The response of *Acanthopagrus schlegelii* to dietary defatted *Hermetia illucens* larvae (HIL) meal irradiated by γ‐rays at various doses was examined. Five test diets containing 80 g/kg crude fat, 420 g/kg crude protein, and 200 g/kg fishmeal were designed. All test diets contained 224 g/kg defatted HIL meal irradiated with γ‐rays at a dose of 0 (D0), 5 (D5), 10 (D10), 20 (D20), or 40 kGy (D40). Compared to fish fed diet D0, fish fed diets D5 and D10 exhibited higher values of final body weight, weight gain, retention efficiencies of carbon, nitrogen, and phosphorus, and hepatic total antioxidant capacity, but lower values of feed intake and feed conversion ratio. Opposite trend was observed for these parameters in fish fed diets D20 and D40. No significant differences were observed in the condition factor, hepatosomatic index, and body contents of moisture, crude protein and lipid, carbon, and phosphorus among different groups. Compared to fish fed diet D0, fish fed diets D5 and D10 exhibited reduced or statistically equivalent waste excretion of carbon, nitrogen, and phosphorus. Fish from groups D20 and D40 exhibited higher Simpson index than fish from group D0. The beta diversity of intestinal bacteria differed between fish fed diets D0, D5, and D10 and those fed diets D20 and D40. A gradual decrease in the populations of the pathogenic bacteria *Ralstonia* and *Pseudomonas* was observed with increasing irradiation intensity. In contrast, the population of the intestinal probiotic bacteria *Achromobacter* increased two to four times. The results demonstrate that the potential of γ‐irradiated defatted HIL meal as a protein ingredient for *Acanthopagrus schlegelii* is irradiation dose dependent. Overall, γ‐irradiation of dietary defatted HIL meal at 5 or 10 kGy is beneficial for *Acanthopagrus schlegelii*.

## 1. Introduction

Fishmeal, as the primary source of high‐quality protein for fish culture, plays a pivotal role in the feed industry, especially for carnivorous fish species [1]. Currently, fishmeal has emerged as a protein ingredient with an inadequate supply and high prices, due to the drastic decline in wild fish stocks [2]. Consequently, alternative proteins produced in a resource‐efficient and sustainable manner are urgently needed [3]. Insects are considered as the most promising and sustainable alternative to fishmeal, primarily because of their relatively short life cycle and the capacity to thrive on a diverse range of substrates, providing high‐quality unsaturated fatty acids and proteins [4]. Eight insect species are currently authorized by the European Union for the use in aquafeeds [5]. Of these, *Hermetia illucens* is considered the most promising, with several developmental stages, including eggs, larvae, pupae, and adults [6]. Until now, only the *Hermetia illucens* from larval and pupal stages are processed into feed ingredients [7–9], with *Hermetia illucens* larvae (HIL) meal being the most commonly employed protein source for fish. The HIL meal has been developed as a feedstuff for its high contents of vitamins, minerals, and protein, as well as balanced amino acids [10, 11]. The HIL meal has been extensively studied as an environmentally sustainable fishmeal substitute in numerous carnivorous fish species, like rainbow trout *Oncorhynchus mykiss* [12, 13], Atlantic salmon *Salmo salar* [14], gilthead sea bream *Sparus aurata* [15–17], and yellowtail *Seriola quinqueradiata* [18]. However, high contents of dietary HIL meal have been demonstrated to reduce fish growth, feed intake, and feed utilization [19, 20]. Although defatted HIL meal, with a greater potential to replace fishmeal than HIL meal [21], has a higher digestible protein and lower fat content, it could still impair fish health and growth performance at high inclusion levels, possibly ascribe to the presence of antinutritional factors like chitin [22]. Therefore, it is of paramount importance to enhance the utilization efficiency of HIL meal in order to optimize its utilization.

Irradiation refers to a process whereby the target material is exposed to ionizing radiation that produced by high‐energy electrons, γ‐ or X‐rays, which can lead to a number of chemical reactions including polymerization, cross‐linking, degradation, and modification of the target substance [23]. The utilization of γ‐irradiation in aquaculture has garnered increasing attention in recent years, with evidence demonstrating its efficacy as a method for the improvement of fish growth, and the enhancement of the potential of alternative proteins to replace dietary fishmeal. For instance, feeding γ‐irradiated palm fruit extracts to goldfish *Carassius auratus* enhanced growth performance, hepatic antioxidant capacity, and skin mucosal immunity [24]. The γ‐irradiated feather meal or soybean meal was found to be more promising as fishmeal substitutes than its nonirradiated counterparts in the diets for golden pompano *Trachinotus ovatus* [25, 26], largemouth bass *Micropterus salmoides* [27, 28], and Japanese sea bass *Lateolabrax japonicas* [29]. Furthermore, our recent study found that γ‐irradiation could improve dietary defatted HIL meal as an alternative to fishmeal for black sea bream *Acanthopagrus schlegelii* [30]. It has also been reported that the functional properties of plant protein sources such as cowpea or sesame flour were affected by γ‐irradiation in a dose‐dependent manner [31, 32]. However, it is still unclear if the potential of γ‐irradiated defatted HIL meal as a fishmeal substitute in fish diets is irradiation dose dependent. Moreover, it has been demonstrated that γ‐irradiation can influence the gut microbiota of terrestrial animals, leading to a healthier state characterized by an increase in probiotic bacteria and a reduction in harmful bacteria [33, 34]. This was verified in our recent study in which it was found that γ‐irradiation improved the growth performance of *Acanthopagrus schlegelii* fed with defatted HIL meal, alongside increased relative abundance of intestinal probiotic bacteria [30]. However, whether the response of intestinal microbiota in *Acanthopagrus schlegelii* to γ‐irradiated defatted HIL meal is dose dependent is still unclear.

*Acanthopagrus schlegelii*, also known as black sea bream, is a highly consumed marine fish in China because of its palatable taste, rapid growth rate, and adaptability to a wide range of salinities and temperatures [35]. As demonstrated by our recent findings that treatment of defatted HIL meal with γ‐rays could reduce dietary fishmeal to 200 g/kg for *Acanthopagrus schlegelii* without any adverse effects on growth performance, body components, and gut bacterial diversity [30]. To elucidate the response of *Acanthopagrus schlegelii* to dietary defatted HIL meal irradiated at different intensities of γ‐rays and to determine the appropriate irradiation dose to improve the potential of defatted HIL meal as a protein source in the diet of *Acanthopagrus schlegelii*, the current study therefore designed a series of diets containing 200 g/kg fishmeal and 224 g/kg defatted HIL meal irradiated with different doses of γ‐rays, to explore the effects of feeding γ‐irradiated defatted HIL meal to *Acanthopagrus schlegelii* on the growth, feed utilization, waste production, antioxidant capacity, and gut microbiota.

## 2. Materials and Methods

### 2.1. Ethics Approval

The fish‐related procedures and sampling were conducted in accordance with the Management Rule of Laboratory Animals (Chinese Order No. 676 of the State Council, revised March 1, 2017).

### 2.2. Treatment of Defatted HIL Meal

The defatted HIL meal produced by Zhejiang Kunwei Agricultural Technology Co., Ltd (Hangzhou, China) was subjected to γ‐ray treatment by a professional institute called Jiaxiang Irradiation Technology Co., Ltd (Hangzhou, China). Briefly, 5 kg of defatted HIL meal was placed in an irradiated metal box and treated with γ‐rays produced by ^60^Co under an average irradiation dose rate of 4.4 kGy/h at room temperature. To produce γ‐irradiated defatted HIL meal with a dose of 5, 10, 20, and 40 kGy, the treatment duration was extended to 1.14, 2.27, 4.55, and 9.09 h, respectively. The proximate composition, amino acid composition, and fatty acid composition of nonirradiated and γ‐irradiated defatted HIL meal was shown in Supporting Information: Tables S1–S3.

### 2.3. Experimental Design and Feed Production

According to our recent work [30], five iso‐protein (420 g/kg crude protein) and iso‐fat (80 g/kg crude fat) diets were formulated, with each diet containing 224 g/kg of defatted HIL meal and 200 g/kg fishmeal. The defatted HIL meal in the abovementioned five diets received γ‐irradiation at a dose of 0, 5, 10, 20, or 40 kGy, respectively, correspondingly named as diets D0, D5, D10, D20, and D40. The primary protein sources for each experimental diet were imported fishmeal (Peru) and poultry by‐product meal (USA), defatted HIL meal, and soybean meal (Table 1).

**Table 1:** Formulation (g/kg) and proximate composition (g/kg) in the test diets.

Ingredients	D0	D5	D10	D20	D40
Steam dried red fishmeal	200	200	200	200	200
Defatted *Hermetia illucens* larvae (HIL) meal	224	0	0	0	0
Defatted HIL meal, irradiated at 5 kGy	0	224	0	0	0
Defatted HIL meal, irradiated at 10 kGy	0	0	224	0	0
Defatted HIL meal, irradiated at 20 kGy	0	0	0	224	0
Defatted HIL meal, irradiated at 40 kGy	0	0	0	0	224
Poultry by‐product meal	60	60	60	60	60
Blood meal	20	20	20	20	20
Soybean meal	120	120	120	120	120
Cottonseed meal	40	40	40	40	40
Rapeseed meal	30	30	30	30	30
Corn gluten meal	25	25	25	25	25
Wheat flour	165	165	165	165	165
Starch, gel	20	20	20	20	20
Zeolite powder	24	24	24	24	24
CaHPO_4_	20	20	20	20	20
Choline chloride	2	2	2	2	2
Vitamin and mineral premix	20	20	20	20	20
Fish oil	30	30	30	30	30
Proximate composition					
Dry matter	929	927	922	921	925
Crude protein	429	418	412	411	417
Crude lipid	76.5	78.7	83.8	80.5	78.0
Ash	132	129	128	128	127
Carbon	401	426	408	402	416
Phosphorus	13.7	15.1	15.0	13.3	15.2

*Note:* Vitamin and mineral premix provides per kg of feed: vitamin A, 8000 IU; vitamin D3, 2000 IU; vitamin E, 100 mg; vitamin K3, 7.5 mg; vitamin B1, 15 mg; vitamin B2, 15 mg; vitamin B6, 12.5 mg; vitamin B12, 0.05 mg; D‐biotin, 0.25 mg; D‐calcium pantothenate, 40 mg; folic acid, 5 mg; niacinamide, 50 mg; vitamin C, 140 mg; inositol, 120 mg; FeSO_4_, 40 mg; CuSO_4_·5H_2_O, 25 mg; MnSO_4_·4H_2_O, 10 mg; ZnSO_4_, 100 mg; MgSO_4_·7H_2_O, 200 mg; CoCO_3_, 0.35 mg; KI, 0.05 mg; Na_2_SeO_3_, 0.3 mg; *C*_14_H_19_NO, 5 mg. Crude protein, crude lipid, ash, carbon, and phosphorus are expressed on as‐feed basis.

Following the methodology employed in Ren et al. [30], the test diets were prepared by grinding, weighing, and thoroughly mixing the feed ingredients, followed by a 15‐min mixing period with tap water (26%) using a Hobart mixer. Subsequently, feed pellets (3 mm in diameter and 5 mm in length) were produced by extruding the mixture using a laboratory extruder and were finally dried at room temperature.

### 2.4. Experimental Fish and Procedures of Feeding

*Acanthopagrus schlegelii* juveniles were procured from a fish hatchery at Ninghai (Ningbo, China) and transported to the farming base of Sanmen Donghang Aquatic Breeding Technology Co., Ltd (Taizhou, China) using a specialized flow‐through truck. On arrival, the juveniles were housed in an indoor net pen (3 m × 3 m × 2 m) fixed in concrete pools and fed a D0 diet four times a day for 2 weeks. Afterwards, the juveniles were deprived of food for 24 h. Subsequently, 375 juvenile fish of similar size (initial body weight [IBW] 2.33 g) were selected, weighed, and divided into 15 experimental net pens (1 m × 1 m × 1.5 m) with a density of 25 fish per pen. The 15 experimental net pens were randomly numbered with three replicates of each test diet. Fish were satisfied, fed twice daily during a period of 8 weeks. Daily monitoring was conducted to measure water temperature and salinity, which ranged 26.3–29.7°C and 28–34 ppt, respectively.

### 2.5. Sample Collection

Following a 24‐h fast, fish from each net pen were collected, weighed collectively, and counted. A total of eight fish were randomly captured from each pen and deeply anesthetized with 90 mg/L MS‐222 (Sigma–Aldrich). Five of them were dissected, and the hindgut (including chyme) was collected for the analysis of intestinal microbiota, and the livers from three fish were collected for detecting biochemical indices. Liver and gut samples were frozen in liquid nitrogen immediately after collection and subsequently transferred to refrigerators set at −80°C for storage. The other three anesthetized fish were individually measured for body mass and length, and then were dissected to measure the liver mass and finally stored at refrigerators set at −20°C for body component analysis. Fish that have undergone anesthetic dissection and are not to be used for sample collection shall be euthanized by immersion in a lethal dose of anesthetic (about 200 mg/L MS‐222).

### 2.6. Analysis of Proximate Composition, Amino Acids, and Fatty Acids

Fish samples were subjected to an autoclave treatment (120°C, 20 min), homogenized, oven‐dried, and powdered prior to chemical analysis. Carbon content in fish samples, test diets, and ingredients was analyzed using a Vario TOC analyzer manufactured by Elementar. The other components, including crude protein (Method 992.23), lipid (Method 922.06), moisture (Method 925.09), ash (Method 923.03), and phosphorus (Method 995.11), were analyzed using the AOAC [36] procedure.

Following the methodology employed in Ren et al. [37], the amino acid composition in non‐irradiated and γ‐irradiated defatted HIL meal was analyzed using an Agilent 1100 liquid chromatography. The samples for chromatography analysis were pretreated with acid hydrolysis. Briefly, 100 mg of sample was weighed into a sealed bottle, and then 10 mL of 6 mol/L hydrochloric acid (containing 1% phenol) was added. The bottle was then filled with nitrogen for 1 min before being sealed. After hydrolysis at 110°C for 22 h, the sample was cooled and diluted with ultrapure water to a final volume of 50 mL, from which 1 mL of the diluted sample was taken and evaporated it to dryness using nitrogen blowing at 95°C. Finally, the sample was dissolved in 1 mL of 0.01 M hydrochloric acid, and then filtered through a 0.45 mm nylon filter for detection.

Following the methodology employed in Ren et al. [37], the fatty acid composition in nonirradiated and γ‐irradiated defatted HIL meal was analyzed using an Agilent 7890–5975 gas chromatography–mass spectrometry (GC–MS) system. The lipid in the defatted HIL meal was extracted using hydrolysis‐extraction method according to the national standard for the determination of fatty acids in food (GB 5009.168–2016, China), with 1 mg undecanoic acid as the internal standard. All fatty acids were qualitatively identified using computerized NIST 11 spectral library database searches, reference to fatty acid standard spectra, and retention times for each fatty acid. The quantitative analysis of fatty acids was performed using the internal standard method. Fatty acid data were converted based on the quantitative analysis results and presented as % of total identified.

### 2.7. Protein Extraction and SDS‐PAGE

The extraction of protein from regular and irradiated defatted HIL meal was referred to Kim et al. [38] with modifications. Briefly, prior to protein extraction, the regular and irradiated defatted HIL meal were ground and defatted using the Soxhlet extraction method. For protein extraction, 20 mg of defatted HIL meal and 500 μL of buffer (containing 0.5 mol/L Tris‐HCl [pH 6.8] 500 μL, glycerol 1 mL, 10% SDS 2 mL, bromophenol blue 0.01 g, DTT 0.16 g, and adding double‐distilled water to 10 mL) were vortexed for 1 min and then centrifuged at 12,000 rpm for 3 min, the supernatant was collected and boiled in a water bath for 10 min.

Protein subunits from regular and irradiated defatted HIL meal were analyzed using SDS‐PAGE electrophoresis. Each sample was extracted with precision and loaded into the sample well on an isonitrogen basis (10 μg). Electrophoresis was performed simultaneously on a 12% acrylamide separating gel and a 5% acrylamide concentrating gel. The SDS gels were then stained with Coomassie Brilliant Blue R‐250.

### 2.8. Analysis of Biochemical Indicators in Liver

Prior to biochemical analysis, samples were defrosted and homogenized in physiological saline (0.86%, 1w:4v), which had been pre‐cooled to 4°C. The homogenate was then centrifuged at 9500 rpm (10 min) using a centrifuge set at 4°C, and subsequently the supernatant was collected in order to detect the MDA content and activities of T‐AOC, SOD, and GSH‐Px, which were measured using commercial kits (Nanjing Jiancheng Bioengineering Institute, Nanjing, China). The analysis was performed using a Thermo enzyme reader.

### 2.9. The DNA Extraction and Gut Microbiology Analysis

Total DNA extraction from gut bacteria and 16S rRNA gene amplification were performed following Ren et al. [30]. Briefly, the extraction of total intestinal bacterial DNA was conducted using a TIANamp Marine Animals DNA Kit (Tiangen) following standardized procedures. DNA integrity and purity were assessed via 1% agarose gel electrophoresis, and DNA concentration and purity were simultaneously measured using the NanoDrop One. The V3–V4 region of the 16S rRNA gene was then amplified utilizing universal primers 338F (5^′^‐ACTCCTACGGAGGCAGCA‐3^′^) and 806R (5^′^‐GGCTACHVGGGTWTCTAAT‐3^′^). The PCR reaction system was set up at 50 μL comprising of 25 μL 2× Premix Taq (Takara Biotechnology, Dalian Co. Ltd., China), 1 μL each primer (10 μM), and 3 μL DNA template (20 ng/μL). The PCR amplification was performed on a BioRad S1000 (Bio‐Rad, USA) starting with 1 cycle of initial denaturation (94°C for 5 min), followed by 30 cycles of denaturation (94°C for 30 s), 1 cycle of annealing (52°C for 30 s), and 1 cycle of extension (72°C for 30 s), concluded with a final extension step (72°C for 10 min). Subsequent library preparation followed the standard procedure of the NEBNext Ultra DNA Library Prep Kit for Illumina. The sequencing of 16S rDNA was carried out using the Illumina Nova 6000 platform by Guangdong Magigene biotechnology Co., Ltd (Shenzhen, China). Sequences with a similarity of ≥97% were grouped into the same operational taxonomic units (OTUs).

### 2.10. Calculation

Equations were calculated in accordance with Ren et al. [30] and presented as follows.

Feed intake % BW/day=100 ×Mfeed / M0+Mt+Md/2×T,

Final body weight FBW, g/fish=Mt/Nt,

Weight gain g/fish=Mt/Nt−M0/N0,

Feed conversion ratio FCR=Mfeed/Mt−M0+Md,

Carbon retention efficiency CRE, %=100 × Mt×CCt−M0×CC0/Mfeed×CCf,

Nitrogen retention efficiency NRE, %=100 × Mt×CNt−M0×CN0/Mfeed×CNf,

Phosphorus retention efficiency PRE, %=100 × Mt×CPt−M0×CP0/Mfeed×CPf,

Carbon waste g C/kg fish gain=1000 × Mfeed×CCf×1100−CRE//Mt−M0,

Nitrogen waste g N/kg fish gain=1000 × Mfeed×CNf×1100−NRE//Mt−M0,

Phosphorus waste g P/kg fish gain=1000 × Mfeed×CPf×1100−PRE//Mt−M0,

Condition factor g/cm3=100×Ms/Lfish3,

Hepatosomatic index %=100×Mliver/Mfish,

where *M*_feed_ (g) represents the total mass of feed consumed, *M*_0_ (g) and *M*_t_ (g) represent the total body mass of fish at the start and end, while *M*_d_ represents the total body mass of death fish, *T*(d) represents the duration of the trial, *N*_0_ refers to the number of fish in each net pen at the beginning of the trial and *N*_t_ is at the end, *C*_Ct_ (%) and *C*_C0_ (%) represent the body content of carbon at the end and start of the trial, respectively. *C*_Nt_ (%) and *C*_N0_ (%) represent the body content of nitrogen at the end and start of the trial, respectively. *C*_Pt_ (%) and *C*_P0_ (%) represent the body content of phosphorus at the end and start of the trial, respectively. *M*_fish_ (g/fish) corresponds to the body mass of fish, *M*_liver_ (g) corresponds to the liver mass of fish, and *L*_fish_ (cm) corresponds to fish body length.

### 2.11. Statistics

Prior to statistical analysis, all data were tested for normality using the one‐sample Kolmogorov–Smirnov test and for homogeneity of variances via Levene’s test. The statistical analysis methods refer to our previously published article [39], namely, data were analyzed utilizing one‐way ANOVA. In cases, where significant intergroup differences were detected, pairwise comparisons of means were further conducted using Tukey’s HSD test. Orthogonal polynomial contrasts were applied to ascertain whether the observed effects exhibited linear or quadratic trend, while regression analysis was employed to estimate the optimal irradiation dose for defatted HIL meal treatment. Analyses including ANOVA, Tukey’s HSD test, and orthogonal polynomial contrasts were executed using professional statistical software (IBM SPSS, version 21.0). All data visualizations were generated using Origin software (version 2021) or the bioinformatics online platform (https://www.omicsmart.com/#/). Statistical significance was defined as *p*  < 0.05.

## 3. Results

### 3.1. Growth, Feed Utilization, and Waste Output

**Figure 1:**
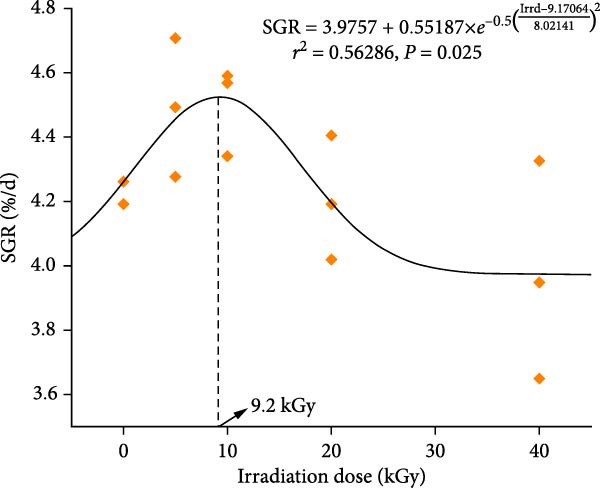
Optimal irradiation dose (Irrd) for treating *Hermetia illucens* larvae meal based on the specific growth rate (SGR) of juvenile black sea bream *Acanthopagrus schlegelii*.

At harvest, the survivals of groups D0–D40 were 80%, 79%, 80%, 71%, and 63%, respectively. The IBW among different groups exhibited no significant difference (*p* > 0.05, Table 2). The highest FBW and weight gain were observed in groups D5 and D10, followed by groups D0 and D20, and the lowest FBW and weight gain were found in group D40 (*p* < 0.05). The highest feed intake was found in group D40, followed by groups D0, D10, and D20, and the lowest feed intake was found in the D5 group (*p* < 0.05). Higher FCR was observed in groups D20 and D40 than in groups D5 (*p* < 0.05). Higher CRE and PRE were found in groups D5 and D10 than in groups D20 and D40 (*p* < 0.05). The highest PRE was found in groups D5, D10, and D20, followed by group D0, and the lowest PRE was found in the D40 group (*p* < 0.05). The highest carbon waste was found in groups D20 and D40, followed by group D0, and the lowest carbon waste was found in groups D5 and D10 (*p* < 0.05). Higher nitrogen waste was found in groups D0, D20, and D40 than in groups D5 and D10 (*p* < 0.05). Lower phosphorus waste was found in groups D0, D5, D10, and D20 than in group D40 (*p* < 0.05). The regression analysis indicated that the optimal γ‐ray irradiation dose for defatted HIL meal was 9.19 kGy, as estimated by the specific growth rate (SGR) fit (Figure 1). Both of linear and quadratic responses were found in the IBW, FBW, weight gain, feed intake, FCR, CRE, NRE, and PRE, with increasing irradiation dose on treating defatted HIL meal.

**Table 2:** Initial body weight (IBW, g/fish), final body weight (FBW, g/fish), weight gain (g/fish), feed intake (%BW/d), feed conversion ratio (FCR), retention efficiencies of carbon (CRE, %), nitrogen (NRE, %) and phosphorus (PRE, %), waste output of carbon (g C/kg fish gain), nitrogen (g N/kg fish gain) and phosphorus (g P/kg fish gain), and survival (%) of juvenile black sea bream *Acanthopagrus schlegelii* fed the test diets.

Parameters	D0	D5	D10	D20	D40	*p*‐Value		
						ANOVA	Linear	Quadratic
IBW	2.31 ± 0.11	2.33 ± 0.14	2.39 ± 0.15	2.27 ± 0.12	2.34 ± 0.13	0.853	0.001	< 0.001
FBW	24.75 ± 0.62^ab^	28.88 ± 2.91^b^	29.67 ± 0.51^b^	24.10 ± 3.63^ab^	21.77 ± 3.14^a^	0.016	0.005	< 0.001
Weight gain	22.44 ± 0.51^ab^	26.55 ± 2.93^b^	27.28 ± 0.65^b^	21.83 ± 3.53^ab^	19.43 ± 3.23^a^	0.016	0.005	< 0.001
Feed intake	3.89 ± 0.17^ab^	3.27 ± 0.07^a^	3.53 ± 0.24^ab^	4.00 ± 0.44^ab^	4.01 ± 0.29^b^	0.027	0.001	< 0.001
FCR	1.22 ± 0.05^ab^	1.01 ± 0.02^a^	1.09 ± 0.07^ab^	1.27 ± 0.13^b^	1.29 ± 0.07^b^	0.006	0.039	0.117
CRE	30.71 ± 2.12^ab^	35.20 ± 2.02^b^	34.38 ± 2.80^b^	28.41 ± 2.43^a^	26.64 ± 0.38^a^	0.002	0.005	0.001
NRE	29.33 ± 1.61^a^	36.27 ± 1.44^b^	34.79 ± 2.27^b^	28.95 ± 2.47^a^	27.26 ± 1.56^a^	0.001	0.004	< 0.001
PRE	45.44 ± 2.58^ab^	50.78 ± 2.62^b^	48.81 ± 1.15^b^	47.97 ± 3.65^b^	36.94 ± 6.81^a^	0.011	0.005	< 0.001
Carbon waste	382.35 ± 29.27^ab^	311.47 ± 3.59^a^	324.21 ± 31.40^a^	419.97 ± 54.53^b^	458.86 ± 25.44^b^	0.001	< 0.001	< 0.001
Nitrogen waste	66.76 ± 4.71^b^	48.07 ± 2.73^a^	52.08 ± 4.69^a^	68.16 ± 8.83^b^	72.97 ± 5.22^b^	0.001	< 0.001	< 0.001
Phosphorus waste	10.25 ± 0.92^a^	8.40 ± 0.74^a^	9.26 ± 0.61^a^	10.09 ± 1.68^a^	14.46 ± 2.03^b^	0.002	< 0.001	< 0.001
Survival	80 ± 4.00	79 ± 16.17	80 ± 0	71 ± 20.53	63 ± 4.62	0.365	0.033	0.111

*Note:* D0: diet contains 22.4% of defatted *Hermetia illucens* larvae (HIL) meal; D5–D40: diet contains 22.4% of defatted HIL meal irradiated at 5, 10, 20, and 40 kGy, respectively. Data are presented as means ± SD. Data in the same row with different superscripts mean significant difference at *p*  < 0.05.

### 3.2. Somatic Index and Body Composition

At the conclusion of the feeding trial, no significant differences were found in condition factor, hepatosomatic index, and whole body contents of moisture, crude protein, crude lipid, carbon, and phosphorus among different groups (*p* > 0.05, Table 3). Compared to the D0 group, higher body ash content was found in groups D20 and D40, while comparable body ash content was found in groups D5 and D10 (*p* < 0.05). Both of linear and quadratic responses were found in the condition factor, hepatosomatic index, and whole body ash content, with increasing irradiation dose on treating defatted HIL meal.

**Table 3:** Condition factor (g/cm^3^), hepatosomatic index (%), and proximate composition (g/kg) of juvenile black sea bream *Acanthopagrus schlegelii* fed the test diets.

Parameters	D0	D5	D10	D20	D40	*p*‐Value		
						ANOVA	Linear	Quadratic
Condition factor	1.83 ± 0.16	1.91 ± 0.13	1.78 ± 0.15	1.82 ± 0.08	1.82 ± 0.15	0.399	<0.001	<0.001
Hepatosomatic index	1.79 ± 0.25	1.69 ± 0.31	1.75 ± 0.45	1.72 ± 0.32	1.67 ± 0.42	0.953	<0.001	<0.001
Moisture	676.72 ± 6.22	673.47 ± 6.90	673.94 ± 1.49	682.30 ± 5.58	674.01 ± 6.92	0.360	0.001	<0.001
Crude protein	170.61 ± 1.94	169.03 ± 1.14	171.13 ± 1.98	169.98 ± 3.31	168.14 ± 1.42	0.449	0.001	<0.001
Crude lipid	96.21 ± 6.62	97.67 ± 7.73	93.76 ± 2.36	87.25 ± 2.81	93.79 ± 6.24	0.267	0.002	<0.001
Ash	48.73 ± 0.58^a^	49.54 ± 1.26^ab^	50.32 ± 1.93^ab^	52.04 ± 0.69^b^	52.12 ± 0.95^b^	0.020	0.001	<0.001
Carbon	162.27 ± 4.83	163.37 ± 13.07	163.27 ± 4.20	158.34 ± 1.90	158.56 ± 5.29	0.811	0.002	<0.001
Phosphorus	8.49 ± 0.25	8.62 ± 0.15	8.78 ± 0.41	9.10 ± 0.15	8.39 ± 1.19	0.669	0.002	<0.001

*Note:* D0: diet contains 22.4% of defatted *Hermetia illucens* larvae (HIL) meal; D5–D40: diet contains 22.4% of defatted HIL meal irradiated at 5, 10, 20, and 40 kGy, respectively. Data are presented as means ± SD. Data in the same row with different superscripts mean significant difference at *p*  < 0.05.

### 3.3. Composition of Protein Subunit of Defatted HIL Meal

**Figure 2:**
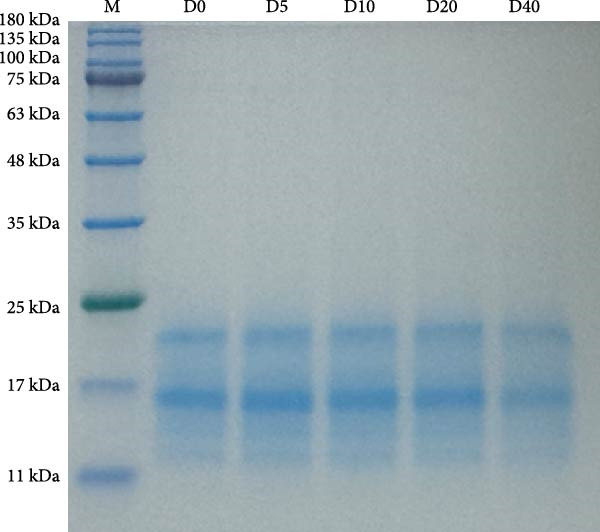
Electrophoretic profiles of proteins in defatted *Hermetia illucens* larvae meal without irradiation (D0) or irradiated at 5 kGy (D5), 10 kGy (D10), 20 kGy (D20), and 40 kGy (D40). Proteins (10 μg) extracted from each sample were electrophoresed in SDS gel and stained by Coomassie Brilliant blue R‐250. The protein marker is shown in lane M.

No difference was observed in the composition of protein subunit (Figure 2) between regular and irradiated defatted HIL meal.

### 3.4. Hepatic Enzymes Activities and MDA Content

The highest T‐AOC activity was found in group D10, followed by that in groups D0 and D5, and the lowest T‐AOC activity was found in groups D20 and D40 (*p* < 0.05, Table 4). The highest SOD activity was found in group D5, followed by groups D0, D10, and D20, and the lowest SOD activity was found in group D40 (*p* < 0.05). The GSH‐Px activity tended to decline with the enhancement of irradiation dose (*p* < 0.05). Higher content of MDA was found in group D40 than in groups D0, D5, and D20 (*p* < 0.05). Both of linear and quadratic responses were found in the activities of T‐AOC and SOD, as well as in MDA content, with increasing irradiation dose on treating defatted HIL meal. However, only quadratic response was found in the GSH‐Px activity (*p* = 0.021).

**Table 4:** Activities of total antioxidant capacity (T‐AOC, U/g prot), superoxide dismutase (SOD, U/mg prot), and glutathione peroxidase (GSH‐Px, U/mg prot), as well as content of malondialdehyde (MDA, nmol/mg prot) in liver of juvenile black sea bream *Acanthopagrus schlegelii* fed the test diets.

Parameters	D0	D5	D10	D20	D40	*p*‐Value		
						ANOVA	Linear	Quadratic
T‐AOC	0.34 ± 0.02^b^	0.35 ± 0.02^b^	0.43 ± 0.02^c^	0.21 ± 0.01^a^	0.20 ± 0.01^a^	<0.001	0.025	0.004
SOD	1882.57 ± 80.14^ab^	1908.59 ± 131.19^b^	1801.95 ± 108.71^ab^	1745.92 ± 119.87^ab^	1601.59 ± 108.39^a^	<0.001	0.003	0.001
GSH‐Px	131.34 ± 1.48^d^	100.18 ± 9.79^c^	81.96 ± 12.36^bc^	65.49 ± 7.60^ab^	48.83 ± 6.17^a^	<0.001	0.053	0.021
MDA	4.54 ± 0.30^a^	4.56 ± 0.31^a^	4.86 ± 0.35^ab^	4.15 ± 0.65^a^	5.85 ± 0.62^b^	0.012	<0.001	<0.001

*Note:* D0: diet contains 22.4% of defatted *Hermetia illucens* larvae (HIL) meal; D5–D40: diet contains 22.4% of defatted HIL meal irradiated at 5, 10, 20, and 40 kGy, respectively. Data are presented as means ± SD. Data in the same row with different superscripts mean significant difference at *p*  < 0.05.

### 3.5. Bacterial Composition and Diversity in Gut

A total of 96,879, 101,655, 98,260, 105,053, and 102,113 sequences were identified for groups D0, D5, D10, D20, and D40, respectively. The sequences were subsequently grouped into 100 (D0), 87 (D5), 78 (D10), 59 (D20), and 88 (D40) OTUs. Only one main phylum (with relative abundance >10%) was detected in all groups: Proteobacteria (Figure 3A), and the relative abundance was 99.02% (D0), 99.22% (D5), 99.10% (D10), 99.47% (D20), and 98.31% (D40). At the genus level (Figure 3B), the predominant bacterial community that has a relative abundance higher than 10% in the D0 group were *Ralstonia* (56.02%), *Achromobacter* (17.86%), and *Pseudomonas* (14.86%). Similarly, *Achromobacter* (45.02%), *Ralstonia* (34.11%), and *Pseudomonas* (10.63%) were also found to be the dominant bacterial genera in the D10 group. On the contrary, *Achromobacter* and *Ralstonia* were the dominant bacterial genera in groups D5, D20, and D40. The relative abundances of *Achromobacter* in groups D5, D20, and D40 were 41.78%, 71.94%, and 71.40%, respectively, and the corresponding relative abundances of *Ralstonia* were 36.77%, 13.53%, and 12.47%. There were no statistically significant differences observed in the relative abundance of bacteria at either the phylum or genus level.

Figure 3The gut microbial composition in juvenile black sea bream *Acanthopagrus schlegelii* fed the test diets at phylum (A) and genus (B) levels. Only the top 10 most abundant (based on relative abundance) bacterial phyla and genera were shown. Other phyla and genera were all assigned as ‘Others’. D0: diet contains 22.4% of defatted *Hermetia illucens* larvae (HIL) meal; D5–D40: diet contains 22.4% of defatted HIL meal irradiated at 5, 10, 20, and 40 kGy, respectively.

**Figure:**
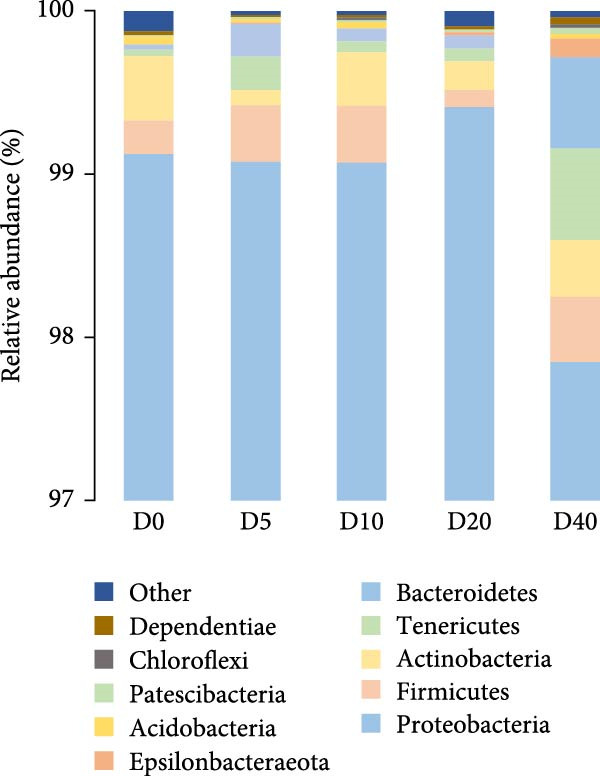
(A)

**Figure:**
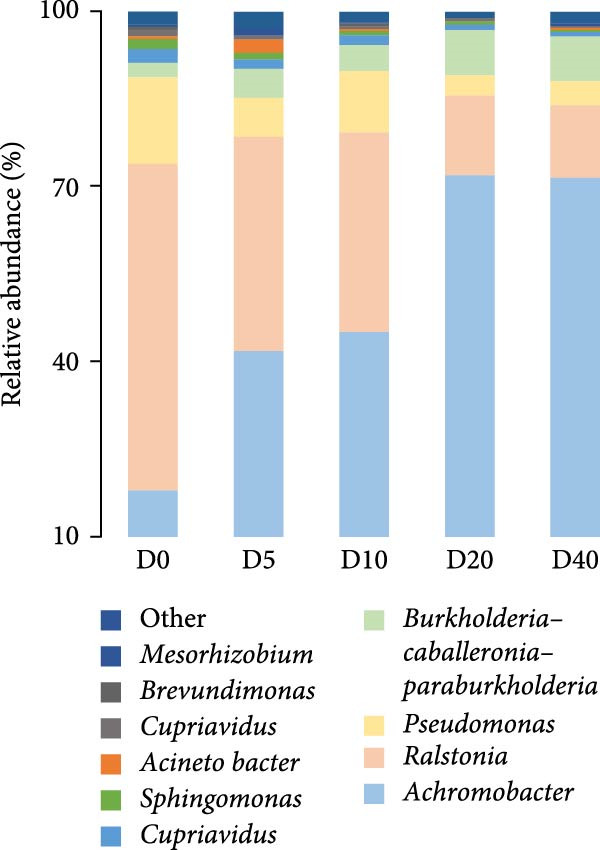
(B)

To ascertain the overlapping and distinctive OTUs among different groups, a Venn diagram was employed (Figure 4A). The analysis revealed that 30 OTUs were shared in common by all groups, while groups D0, D5, D10, D20, and D40 exhibited 39, 23, 29, 26, and 30 unique OTUs, respectively. Moreover, group D0 displayed the highest OTUs, whereas group D20 demonstrated the lowest OTUs. A heatmap was utilized to illustrate the clustering of the top 37 OTUs in the gut bacteria among fish fed distinct test diets (Figure 4B). Heatmap analysis demonstrated that the top 37 OTUs exhibited a considerable degree of variability across different treatments.

Figure 4The overlapping and distinctive OTUs based on Venn diagram (A) and clustering based on the top 37 OTUs (B) in intestinal microbial community of juvenile black sea bream *Acanthopagrus schlegelii* fed the test diets. D0: diet contains 22.4% of defatted *Hermetia illucens* larvae (HIL) meal; D5–D40: diet contains 22.4% of defatted HIL meal irradiated at 5, 10, 20, and 40 kGy, respectively.

**Figure:**
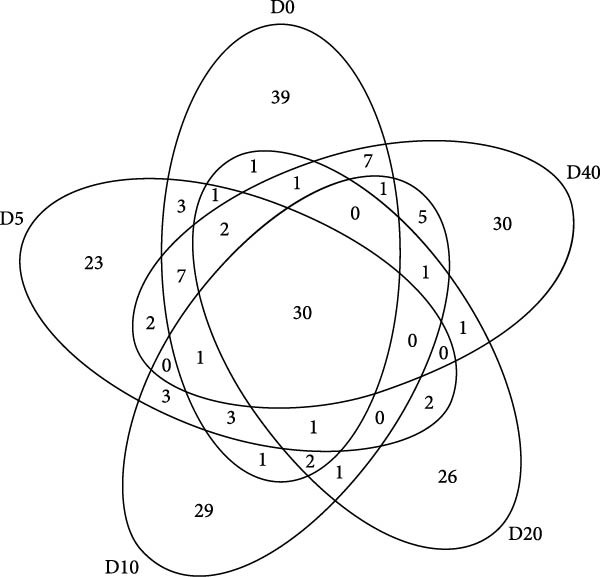
(A)

**Figure:**
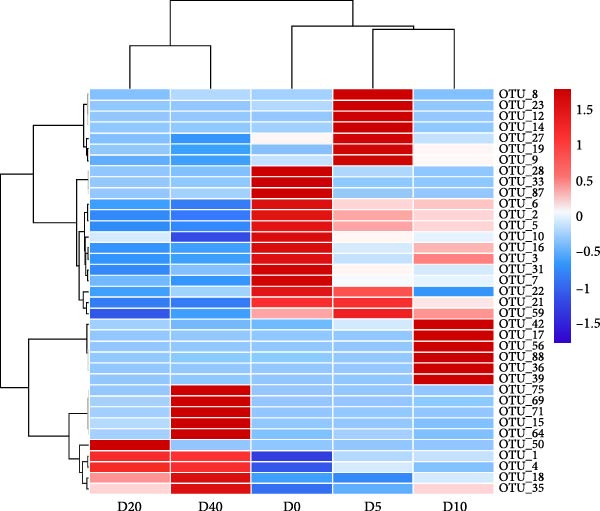
(B)

Two alpha diversity metrics (Chao1, Simpson) and three beta diversity metrics (PCA, NMDS, and UPGMA) were employed in our study. Chao1 focus on the species richness in the sample, and Simpson index incorporate both species richness and evenness. Fish from group D0 exhibited the highest Chao 1, followed by fish from groups D10, D20, and D40, and the lowest Chao 1 were found in group D5 (*p* < 0.05, Figure 5A). The highest Simpson index was found in groups D20 and D40, followed by group D10, and the lowest Simpson index was found in groups D0 and D5 (*p* < 0.05, Figure 5B). The results of principal component analysis (PCA) showed that the PCA1 explained 8.8% of the total variations, while PCA2 explained 7.3% (Figure 5C). The PCA results showed that the samples in all groups were distributed along the direction of the combined PC1 and PC2. The distributions of the D5 and D10 groups exhibited well overlap with those of the D0 group, compared to the overlap between group D0 and group D20 or D40. Results of bray_curtis NMDS showed that the samples in groups D5 and D10 were overlapped with that from group D0, while the samples in groups D20 and D40 were far away from that in group D0 (Figure 5D). The UPGMA results demonstrated that the distance between group D0 and group D5 or D10 was short (Figure 5E).

Figure 5Alpha and beta diversity of the intestinal microbiota in juvenile black sea bream *Acanthopagrus schlegelii* fed the test diets. (A) Chao1 index; (B) Simpson diversity index; (C) Principal component analysis (PCA) based on OTUs levels; (D) Bray_curtis NMDS; (E) UPGMA clustering tree. D0: diet contains 22.4% of defatted *Hermetia illucens* larvae (HIL) meal; D5–D40: diet contains 22.4% of defatted HIL meal irradiated at 5, 10, 20, and 40 kGy, respectively.

**Figure:**
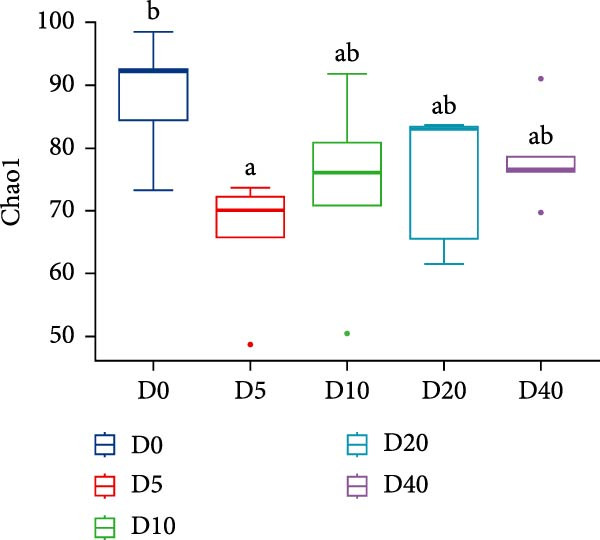
(A)

**Figure:**
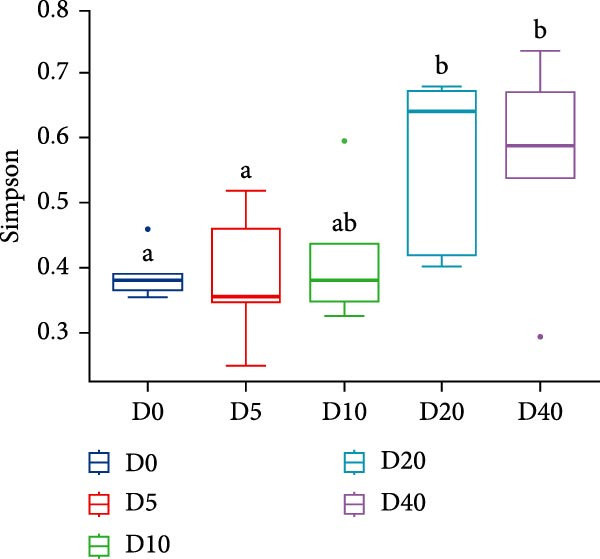
(B)

**Figure:**
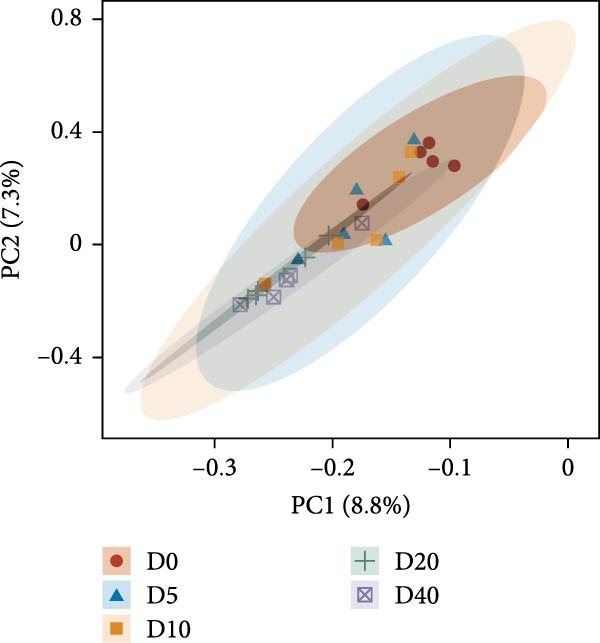
(C)

**Figure:**
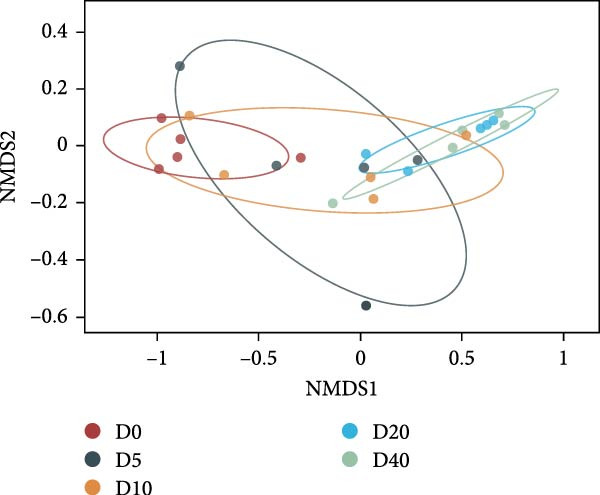
(D)

**Figure:**
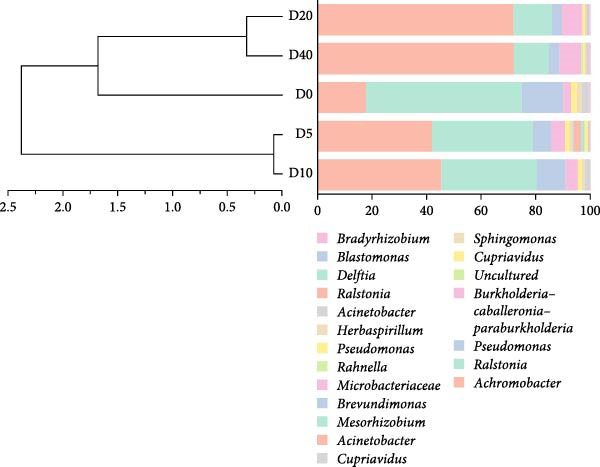
(E)

## 4. Discussions

Apart from one strategy for reducing anti‐nutritional factors in protein ingredients for animals [40] and eliminating allergens in food proteins [41], γ‐irradiation has also been shown to be an effective method for improving growth and feed utilization in fish. For instance, in Japanese sea bass and golden pompano fed an 80 g/kg fishmeal‐based diet, the use of γ‐irradiated soybean meal resulted in higher FBW, weight gain, NRE, and PRE, while lower FCR compared with the inclusion of regular soybean meal [26, 29]. In largemouth bass and golden pompano fed a diet containing 150 g/kg fishmeal, dietary employment of γ‐irradiated feather meal yielded superior FBW, weight gain, and PRE than the inclusion of regular feather meal [27, 42]. In goldfish fed a 180 g/kg fishmeal‐based diet, the utilization of γ‐irradiated *Phoenix dactylifera* fruit extract increased the FBW and SGR and decreased the FCR than that inclusion of nonirradiated *Phoenix dactylifera* fruit extract [24]. In *Acanthopagrus schlegelii* fed a 200 g/kg fishmeal‐based diet, the incorporation of γ‐irradiated defatted HIL meal increased FBW, weight gain, and NRE, while lowered FCR compared with the inclusion of nonirradiated defatted HIL meal [30]. In the current study, in comparison with the D0 group, groups D5 and D10 displayed higher values of FBW, weight gain, CRE, NRE, and PRE, but lower values of feed intake and FCR. However, the opposite trend was observed for these parameters in fish from groups D20 and D40. These findings indicate that the incorporation of dietary defatted HIL meal treated with γ‐rays at a dose of 5 or 10 kGy can enhance the growth performance and feed utilization efficiency of *Acanthopagrus schlegelii*, whereas the utilization of dietary defatted HIL meal treated with γ‐rays at a dose of 20 or 40 kGy has a negative effect. Furthermore, the regression analysis of the SGR fit in our study reveals that the optimal irradiation dose for the treatment of defatted HIL meal in diets for *Acanthopagrus schlegelii* is 9.2 kGy, which is close to the maximum safe dose (10 kGy) of irradiation on food adopted by the Codex Alimentarius Commission [43, 44].

Physical indices are crucial in assessing the health of fish. It is widely accepted that fat accumulation or inflammation can significantly affect visceral function and nutrient metabolism, with notable changes in condition factor and hepatosomatic index [42]. In this study, no significant differences were observed in condition factor and hepatosomatic index among different groups, suggesting that treatment of dietary defatted HIL meal with γ‐rays does not affect the physical health of *Acanthopagrus schlegelii*. Additionally, *Acanthopagrus schlegelii* fed with different test diets exhibited statistically equal body contents of moisture, crude protein, crude lipid, carbon, and phosphorus. Similar to our study, feeding some other carnivorous fish species with γ‐irradiated feed protein ingredients does not influence the physical indices and whole body components. For instance, feeding γ‐irradiated soybean meal to Japanese sea bass [29], γ‐irradiated feather meal to largemouth bass [27], and γ‐irradiated soybean meal or corn gluten meal to golden pompano [45, 46] did not affect the condition factor, hepatosomatic index, and body contents of moisture, crude protein, and lipid.

Fish have an antioxidant defense system comprising antioxidant enzymes and small molecule scavengers [47]. The overall antioxidant status in fish body, representing the level of nonenzymatic and enzymatic original antioxidants, can be quantified by T‐AOC [48]. Among the antioxidant enzymes, SOD detoxifies superoxide anions, while GSH‐Px is a scavenger of hydrogen peroxide [49]. If the free radicals cannot be scavenged in time by the antioxidant defense system, MDA will be generated as an end product of lipid peroxidation [50]. In the current study, a gradual decrease in hepatic activity of SOD and GSH‐Px was observed with increasing irradiation intensity, while for the hepatic T‐AOC activity, it appeared to increase initially and then decrease, with a peak value occurring in group D10. These results indicate that treatment of dietary defatted HIL meal with γ‐rays at 10 kGy is beneficial for the overall antioxidant status in the liver of *Acanthopagrus schlegelii*, although treatment of dietary defatted HIL meal with γ‐rays decreases the activities of SOD and GSH‐Px. It is crucial to note that a multiple of antioxidant enzymes and antioxidants are present in organisms. As a result, the trend of T‐AOC may not always coincide with the trend of certain indicators, such as SOD and GSH‐Px [51]. Therefore, in order to assess the overall status of the antioxidant defense system in *Acanthopagrus schlegelii*, it is essential to take into account changes in downstream indicators, such as inflammatory response and oxidative damage. Compared with the hepatic MDA content in fish fed diet D0, fish fed diet D40 showed a significant increase, while no statistical difference was found in fish fed diets D5, D10, or D20. These results indicate that the lipid peroxidation in the liver of *Acanthopagrus schlegelii* will not accelerate unless the dietary defatted HIL meal is treated with γ‐rays at a dose exceeding 20 kGy.

Waste discharges of carbon, nitrogen, and phosphorus are key indicators for quantifying the environmental impact of fish aquaculture, as they are the main drivers of eutrophication in water bodies [42, 52]. In our study, fish from groups D5 and D10 exhibited reduced or statistically equivalent waste excretion of carbon, nitrogen, and phosphorus compared to fish from group D0. Our results show that feeding *Acanthopagrus schlegelii* with HIL meal irradiated with a suitable dose of γ‐rays does not increase the waste production of carbon, nitrogen, and phosphorus during the aquaculture of this species. Similar outcomes were observed in other carnivorous fish species fed diets incorporating γ‐irradiated feed ingredients. To illustrate, the incorporation of γ‐irradiated soybean meal into the diet of Japanese sea bass and γ‐irradiated feather meal into the diet of largemouth bass or golden pompano does not result in significant alterations to the excretion of carbon, nitrogen, and phosphorus [25, 27, 41]. Feeding γ‐irradiated soybean meal, soy protein concentrate, or corn gluten meal to golden pompano and feeding γ‐irradiated soybean meal to largemouth bass result in a reduction of phosphorus waste output [26, 28, 46].

Numerous studies have reported a potential association between the gut microbiota and various physiological processes in the host, including nutrient absorption and metabolism, energy expenditure, and immune regulation [53]. Furthermore, the composition of gut bacteria is primarily influenced by the food ingested by the host [54]. Consequently, alterations in the gut bacterial community are commonly employed as an indicator to reflect the host’s physiological response to food. It has been reported that Firmicutes, Proteobacteria, Actinobacteria, and Bacteroidetes are the primary gut bacterial phyla in fish, regardless of food [55]. Similarly, Proteobacteria, Firmicutes, Actinobacteria, Tenericutes, and Bacteroidetes were the five most abundant phyla observed in all groups in our study, especially Proteobacteria, which accounted for up to 99% of the total. Studies have demonstrated that the colonization of *Achromobacter* genus can enhance the antioxidant capacity and reduce oxidative damage in fish intestine [56, 57]. Moreover, it has been reported that the *Achromobacter* genome contains gene clusters associated with polysaccharide cleavage enzymes and glycoside hydrolases [58, 59]. For bacteria genus *Ralstonia* and *Pseudomonas*, they were generally considered opportunistic pathogens that caused bacterial diseases in fish [21, 60]. In the current study, a gradual decline in the populations of *Ralstonia* and *Pseudomonas* was observed with increasing irradiation intensity. However, the population of intestinal *Achromobacter* increased two to four times. These results imply that treating dietary defatted HIL meal with γ‐rays decreases the relative abundance of pathogenic bacteria such as *Ralstonia* and *Pseudomonas* within *Acanthopagrus schlegelii* gut, while simultaneously increasing the relative abundance of *Achromobacter* that can promote the antioxidant capacity and carbohydrate utilization in the host. In accordance with our findings, the administration of polysaccharides extracted from γ‐irradiated *Astragalus* or *Schizophyllum commune* to broilers and mice resulted in an increase in the relative abundance of intestinal probiotic bacteria [33, 34]. Collectively, it can be stated that γ‐irradiation treatment on dietary feed ingredients or additives results in a shift in the composition of the intestinal microbiota in animals towards a healthier state.

It is generally accepted that high levels of richness, evenness, and diversity in the gut microbiota are beneficial for host health [61], given that the gut bacterial community coevolves with their host and closely influences host health [62]. Alpha diversity is employed to quantify the species evenness and richness. Beta diversity, on the other hand, is utilized to assess the discrepancies in microbiota composition between individuals [63]. In this study, two alpha diversity metrics, namely Chao1 and Simpson, were utilized to assess the abundance and species diversity of the intestinal bacterial community, respectively. No statistical difference was detected in Chao1 in fish from groups D20 and D40 compared to fish from group D0. On the contrary, fish from the D20 and D40 groups exhibited higher Simpson index than fish from the D0 group. The above results demonstrate that γ‐irradiation treatment of dietary defatted HIL meal at a dose of 20 or 40 has no negative effects on the abundance of the intestinal microbiota in *Acanthopagrus schlegelii*, while increasing the species diversity. In accordance with our findings, the gut bacterial species diversity also increased in broilers fed with γ‐irradiated *Astragalus* polysaccharides at a dose of 25 kGy [33]. Three beta diversity metrics, PCA, NMDS, and UPGMA clustering tree, were utilized in our study to assess microbial differences between samples and meanwhile visualize trends in their distribution. The results of PCA and NMDS showed that the distribution of the replicates from groups D5 and D10 was close to that of group D0, whereas the distribution of the replicates from groups D20 and D40 exhibited a greater degree of separation from group D0. UPGMA clustering showed that groups D5 and D10 were more similar to group D0 in terms of species composition than groups D20 and D40. These results imply that irradiation of defatted HIL meal with low dose (5 or 10 kGy) of γ‐rays produces comparable beta diversity in the intestinal bacteria of *Acanthopagrus schlegelii*. However, high dose (20 or 40 kGy) of γ‐rays results in substantial changes in the beta diversity of the intestinal bacteria.

The mechanisms by which γ‐ray irradiation enhances the nutritional quality or functional properties of feed ingredients, and how these mechanisms translate into improved growth performance in fish, remain unclear to date. In our study, the ∑PUFA and ∑*n*−6 PUFA values in groups D5, D10, and D20 were much higher than in groups D0 and D40. However, the change in the fatty acid composition of the defatted HIL meal did not fully align with the change in growth performance. Additionally, gamma irradiation had no effect on the protein molecular structure and amino acid profile of the defatted HIL meal. Similar results were found in soybean meal [29] and fishmeal [64] subjected to gamma irradiation. Previous studies have reported that the administration of γ‐irradiated feed additives increases the relative abundance of intestinal probiotic bacteria in other animals. Specifically, broilers fed with γ‐irradiated *Astragalus* extract for 21 days had a higher average daily gain and relative abundance of beneficial gut microbiota like *Shuttleworthia* and *Sellimonas*, than those fed with nonirradiated *Astragalus* extract [33]. Similarly, feeding mice with γ‐irradiated *Schizophyllum commune* extract for 30 days promoted the growth of beneficial gut microbiota (*Akkermansiaceae*, *Lachnospiraceae* and *Bacteroidaceae*), while inhibiting the growth of harmful bacteria (*Muribaculaceae* and *Lactobacillaceae*), compared to the normal *Schizophyllum commune* extract [34]. In this study, the intestinal probiotics increased and the potential pathogenic bacteria declined as the γ‐rays dose enhanced, exhibiting an inconsistent trend in black sea bream growth. These results indicate that alterations in the nutrient content of defatted HIL meal or in the microbiota composition in the fish intestine are not the primary cause of the beneficial effects of γ‐rays on the growth of black sea bream. Further investigation is required to elucidate the underlying mechanisms of these effects when feeding *Acanthopagrus schlegelii* with defatted HIL meal irradiated at different doses of γ‐rays.

## 5. Conclusions

In conclusion, the response of *Acanthopagrus schlegelii* to dietary γ‐irradiated defatted HIL meal is doses‐dependent. Specifically, irradiation of dietary defatted HIL meal with low doses of γ‐rays (5 or 10 kGy) is beneficial for the growth, feed utilization, and hepatic total antioxidant capacity of *Acanthopagrus schlegelii*. However, high doses of γ‐rays (20 or 40 kGy) have a negative effect. Treatment of dietary defatted HIL meal with γ‐rays has no negative effect on the physical indices and whole body components of *Acanthopagrus schlegelii*. Moreover, feeding γ‐irradiated defatted HIL meal to *Acanthopagrus schlegelii* promotes a favorable microbial composition and structure, as evidenced by a downward in the relative abundance of pathogenic bacteria such as *Ralstonia* and *Pseudomonas* and an upward in the relative abundance of potentially beneficial bacteria like *Achromobacter*. The recommended dose of γ‐ray irradiation for dietary defatted HIL meal in *Acanthopagrus schlegelii* is 5 or 10 kGy, which was estimated to be 9.2 kGy through regression analysis on the SGR. It should be noted that due to the absence of data on feed digestibility trials, the detection of anti‐nutritional factors such as chitin content in the feed, and the detailed chemical changes in defatted HIL meal before and after irradiation treatment, the mechanism by which γ‐irradiated defatted HIL meal improves growth performance in *Acanthopagrus schlegelii* remains to be investigated.

## Conflicts of Interest

The authors declare no conflicts of interest.

## Funding

This study was supported by Taizhou Science and Technology Plan Projects (Grants 23nya03 and 24nya22).

## Supporting Information

Additional supporting information can be found online in the Supporting Information section.

## Supporting information


**Supporting Information** Table S1: Proximate composition (%) of the feed ingredients; Table S2: Fatty acid composition (%) of defatted *Hermetia illucens* larvae meal; Table S3: Amino acid composition (mg/g) of defatted *Hermetia illucens* larvae meal.

## Data Availability

The data that supports the findings of this study are available in the tables, figures, and supporting information of this article.
