# Supplementary material for: Treatment of Dietary Defatted Hermetia illucens Larvae Meal With Different Doses of γ‐Rays: Effects on Growth, Waste Production, Antioxidant Capacity, and Gut Microbiota in Acanthopagrus schlegelii
Source: Aquac Nutr. 2026 Jan 15;2026:7228323. doi: 10.1155/anu/7228323 (PMC12808818; doi:10.1155/anu/7228323)
Supplement: Supplementary file 1 — Supporting Information Table S1: Proximate composition (%) of the feed ingredients; Table S2: Fatty acid composition (%) of defatted Hermetia illucens larvae meal; Table S3: Amino acid composition (mg/g) of defatted Hermetia illucens larvae meal. [file ANU-2026-7228323-s001.doc]

**TABLE S1:** Proximate composition (%) of the feed ingredients.

| Ingredients | Dry matter | Crude protein | Crude lipid | Phosphorus |
| --- | --- | --- | --- | --- |
| Defatted HIL meal | 95.56 | 56.53 | 7.77 | 0.82 |
| Defatted HIL meal, 5 kGy | 95.45 | 56.94 | 7.07 | 0.83 |
| Defatted HIL meal, 10 kGy | 96.43 | 55.99 | 8.20 | 0.82 |
| Defatted HIL meal, 20 kGy | 96.03 | 56.56 | 7.58 | 0.81 |
| Defatted HIL meal, 40 kGy | 95.74 | 57.49 | 7.36 | 0.82 |
| Fish meal | 92.64 | 65.33 | 8.21 | 0.13 |
| Poultry by-product meal | 94.34 | 67.22 | 12.55 | 2.40 |
| Blood meal | 94.02 | 88.79 | 0.54 | 0.22 |
| Soybean meal | 89.50 | 44.30 | 1.80 | 0.65 |
| Cottonseed meal | 85.61 | 49.24 | 0.97 | 2.98 |
| Rapeseed meal | 88.23 | 38.44 | 3.63 | 0.23 |
| Corn gluten meal | 87.9 | 79.88 | 1.31 | 0.70 |
| Wheat flour | 86.41 | 14.08 | 1.66 | 0.15 |

HIL: *Hermetia illucens* larvae.

Crude protein, crude lipid and phosphorus are expressed on the ingredient basis (n = 2).

**TABLE S2:** Fatty acid composition (%) of defatted *Hermetia illucens* larvae meal.

|  | DHILM | DHILM, 5 kGy | DHILM, 10 kGy | DHILM, 20 kGy | DHILM, 40 kGy |
| --- | --- | --- | --- | --- | --- |
| C7:0 | 0.036 | 0.013 | 0.028 | 0.031 | 0.033 |
| C8:0 | 0.091 | 0.045 | 0.076 | 0.079 | 0.105 |
| C9:0 | 0.094 | 0.083 | 0.088 | 0.090 | 0.133 |
| C10:1，n6c | 0.023 | 0.044 | 0.041 | 0.027 | 0.013 |
| C10:0 | 1.513 | 1.450 | 1.544 | 1.468 | 1.557 |
| C12:1，n7c | 0.053 | 0.039 | 0.043 | 0.044 | 0.054 |
| C12:0 | 22.921 | 21.556 | 23.539 | 19.650 | 23.788 |
| C13:0 | 0.016 | 0.013 | 0.013 | 0.014 | 0.020 |
| 12Methyl C13:0 | 0.033 | 0.010 | 0.007 | 0.012 | 0.017 |
| C14:1，n5c | 0.576 | 0.377 | 0.397 | 0.380 | 0.441 |
| C14:1，n3c | 0.116 | 0.096 | 0.097 | 0.087 | 0.103 |
| C14:0 | 4.389 | 3.522 | 3.913 | 3.259 | 4.438 |
| 13Methyl C14:0 | 0.021 | 0.025 | 0.018 | 0.023 | 0.030 |
| 12Methyl C14:0 | 0.070 | 0.081 | 0.067 | 0.067 | 0.101 |
| C15:1，n5c | 0.023 | 0.019 | 0.015 | 0.017 | 0.027 |
| C15:0 | 0.147 | 0.120 | 0.127 | 0.125 | 0.157 |
| 14Methyl C15:0 | 0.045 | 0.041 | 0.041 | 0.043 | 0.051 |
| C16:2，n6c | 0.109 | 0.249 | 0.196 | 0.211 | 0.066 |
| C16:1，n7c | 1.102 | 0.931 | 1.054 | 0.935 | 1.078 |
| C16:1，n5c | 2.680 | 2.038 | 2.064 | 2.121 | 2.541 |
| C16:0 | 18.960 | 15.111 | 16.315 | 15.982 | 19.647 |
| 15methyl C16:0 | 0.091 | 0.033 | 0.046 | 0.042 | 0.160 |
| 14methyl C16:0 | 0.116 | 0.148 | 0.109 | 0.125 | 0.122 |
| C17:1，n7c | 0.215 | 0.213 | 0.225 | 0.225 | 0.213 |
| C17:0 | 0.310 | 0.223 | 0.235 | 0.252 | 0.299 |
| C18:2，n6c | 3.304 | 22.960 | 18.685 | 23.230 | 2.544 |
| C18:1，n9c | 27.718 | 15.779 | 17.438 | 16.173 | 26.593 |
| C18:3，n3c | 1.384 | 1.670 | 1.299 | 1.291 | 1.468 |
| C18:1，n9t | 0.330 | 0.402 | 0.300 | 0.465 | 0.411 |
| C18:0 | 5.692 | 4.964 | 5.196 | 5.777 | 5.877 |
| C19:0 | 0.021 | 0.026 | 0.023 | 0.028 | 0.022 |
| C20:4，n6c | 0.593 | 0.684 | 0.509 | 0.755 | 0.600 |
| C20:5，n3c | 0.077 | 0.327 | 0.157 | 0.250 | 0.078 |
| C20:3，n6c | 0.242 | 0.206 | 0.261 | 0.243 | 0.311 |
| C20:2，n6c | 0.601 | 0.417 | 0.593 | 0.567 | 0.673 |
| C20:1，n9c | 0.140 | 0.082 | 0.084 | 0.076 | 0.118 |
| C20:3，n3c | 0.263 | 0.188 | 0.209 | 0.212 | 0.282 |
| C20:0 | 0.531 | 0.485 | 0.535 | 0.600 | 0.610 |
| C21:0 | 0.027 | 0.040 | 0.033 | 0.050 | 0.025 |
| C22:1，n9c | 0.240 | 0.139 | 0.140 | 0.165 | 0.210 |
| C22:0 | 0.424 | 0.451 | 0.521 | 0.629 | 0.551 |
| C23:0 | 0.096 | 0.032 | 0.020 | 0.046 | 0.043 |
| C24:0 | 0.126 | 0.118 | 0.131 | 0.166 | 0.133 |
| ∑SFA | 55.770 | 48.590 | 52.625 | 48.558 | 57.919 |
| ∑UFA | 39.789 | 46.860 | 43.807 | 47.474 | 37.824 |
| ∑MUFA | 33.216 | 20.159 | 21.898 | 20.715 | 31.802 |
| ∑PUFA | 6.573 | 26.701 | 21.909 | 26.759 | 6.022 |
| ∑n-3 PUFA | 1.724 | 2.185 | 1.665 | 1.753 | 1.828 |
| ∑n-6 PUFA | 4.849 | 24.516 | 20.244 | 25.006 | 4.194 |

Fatty acid data were presented on a dry matter basis (n = 2).

**TABLE S3:** Amino acid composition (mg/g) of defatted *Hermetia illucens* larvae meal .

|  | Asp | Glu | Ser | His | Gly | Thr | Arg | Ala | Tyr | Cys | Val | Met | Phe | Ile | Leu | Lys | Pro |
| --- | --- | --- | --- | --- | --- | --- | --- | --- | --- | --- | --- | --- | --- | --- | --- | --- | --- |
| DHILM | 32.42 | 42.92 | 15.88 | 9.26 | 20.94 | 13.09 | 13.40 | 20.65 | 17.45 | 1.10 | 21.85 | 5.49 | 11.04 | 10.73 | 22.24 | 16.64 | 21.44 |
| DHILM, 5 kGy | 33.48 | 45.15 | 16.27 | 9.92 | 19.12 | 13.72 | 13.73 | 21.17 | 19.12 | 1.15 | 22.69 | 5.68 | 6.00 | 11.00 | 11.83 | 12.97 | 17.99 |
| DHILM, 10 kGy | 36.32 | 47.63 | 17.33 | 10.43 | 20.62 | 14.45 | 14.63 | 22.40 | 20.63 | 1.42 | 23.74 | 6.17 | 12.33 | 11.52 | 25.12 | 14.81 | 20.92 |
| DHILM, 20 kGy | 32.33 | 41.65 | 15.62 | 9.37 | 19.52 | 13.00 | 13.14 | 20.38 | 17.93 | 1.06 | 21.61 | 5.34 | 11.05 | 10.66 | 22.23 | 14.40 | 17.93 |
| DHILM, 40 kGy | 33.94 | 42.34 | 15.61 | 9.16 | 20.49 | 13.08 | 13.24 | 20.38 | 18.10 | 0.91 | 21.80 | 5.29 | 11.21 | 10.82 | 22.44 | 15.67 | 21.83 |

Amino acid data were presented on a dry matter basis (n = 2).
